# A Mixed Method Study Exploring Children and Young People's Perception of Energy Drinks and Analysing Consumption Patterns

**DOI:** 10.1111/jhn.70140

**Published:** 2025-10-27

**Authors:** Grace Stewart, Amelia A. Lake, Helen J. Moore

**Affiliations:** ^1^ School of Health and Life Sciences Teesside University Middlesbrough UK; ^2^ Fuse, The Centre for Translational Research in Public Health Newcastle University Newcastle Upon Tyne UK; ^3^ School of Social Sciences Humanities and Law Teesside University Middlesbrough UK

**Keywords:** children, energy drinks, PRIME, young people

## Abstract

**Background:**

Sales and consumption of energy drinks (EDs) continue to rise, especially amongst young people, yet the reason remains unclear. In 2018, a campaign by Jamie Oliver resulted in a voluntary sales ban to under 16‐year‐olds. Also in 2018, the Department of Health and Social Care ran a consultation to end the sales of EDs to under 16 s in England. In the Kings Speech (July 2024), the incoming Labour Government promised to restrict the sale of energy drinks to children. The aim of this mixed method study is to understand children and young people′s perceptions of EDs in North East England UK, post the 2018 voluntary ban and to analyse consumption patterns via focus groups and a survey.

**Methods:**

Fifty students from Years 5 and 6 (*n* = 38 aged 9–11 years) and Year 9 (*n* = 12, aged 13–14 years) were involved in eight semi‐structured focus groups exploring perceptions of EDs; and twenty‐two Year nine students (aged 13–14 years) were involved in a survey measuring consumption of EDs and reasons for use.

**Results:**

This study presents a complex picture of children perceptions of EDs and the emergence of a new popular hydration drink, defined as those marketed as replenishing electrolytes, having low(er) sugar, and improving functional wellness. The survey reported that 81.8% consume EDs. More males consumed EDs in comparison to females and most consumed EDs 2–4 days a week. Qualitative analysis showed factors influencing consumption included marketing, brand association, taste, influence of friends and family, cost and easy access.

**Conclusion:**

Children and young people had a strong understanding around the use of branding and marketing as a powerful tool to encourage purchasing behaviours.

## Summary

- We explore how children and young people (aged 9–14 years) in North East England perceive energy drinks following the UK′s 2018 voluntary ban, using both focus groups and a survey.
- An understanding, among children, regarding energy drinks was observed, which included consumption habits, perceived benefits and risks, and influence of marketing and how they are marketed to.
- The work adds to the literature on dietary influences on children and young people. It offers insights that could inform policy strategies to reduce energy drink consumption in this population.

## 1 Introduction

Energy drinks (EDs) are nonalcoholic beverages that typically contain high amounts of caffeine and sugar in combination with other stimulant ingredients [1, 2]. Ingredients including guarana, taurine, cocoa and yerba mate can be added [2, 3, 4]. Caffeine is one of the world′s most widely consumed psychoactive stimulants; moderate amounts, up to 400 mg can increase alertness, energy and ability to concentrate in adults [5]. Research suggests caffeine can also cause adverse effects which include, but are not limited to; insomnia, restlessness, anxiety, gastrointestinal disturbances, and nausea [6, 7, 8, 9]. In 2015 the European Food Safety Authority (EFSA) proposed a safe habitual caffeine consumption by children and adolescents of 3 mg/kg body weight per day [10]. Hydration drinks have carved out a distinct but related niche within the energy drink market. While traditional energy drinks have high caffeine and sugar content, hydration drinks are marketed as replenishing electrolytes, having low(er) sugar, and improving functional wellness.

Evidence associates EDs with negative physical and mental health in children and young people [11, 12, 13, 14, 15]. Research has stated that EDs have no role in the diets of children or adolescents, as they offer no health benefit, and many of the ingredients are still understudied and unregulated [16, 17, 18]. The caffeine from ED's interferes with the absorption of calcium within the adolescent body during a vital period of maximum bone deposition [19]. The sugar content of EDs typically ranges between 9 and 13 g/100 mL [11]. The consumption of sugar‐sweetened beverages is linked with the prevalence of obesity, heart disease, type 2 diabetes, dental erosion, and some cancers [20, 21, 22, 23, 24, 25]. The caffeine content in EDs ranges from 6 to 242 mgs per serving, however, in reality this is often higher with some ED containers holding more than one serving [26].

### 1.1 Consumption of EDs

In the UK, ED consumption is growing rapidly and young people aged 10–17 years are the highest consumers [27]. A recent analysis reported that up to 32% of young people across the UK, and up to half of children worldwide, consumed EDs weekly [28]. In 2024, the UK ED market was worth £4 billion, and is forecast to increase by another £1 billion by 2029 [29] yet the attractiveness to young people has not been fully explored. Easy access, poor dietary choices, and cost of EDs has been identified in earlier research [26, 27, 30]. Recent evidence found young people who drank EDs on five or more days weekly, were more likely to have reduced physical and psychological health as well as issues with education and well‐being [28]. Studies found that frequent ED consumers were more likely to report health‐compromising behaviours such as substance use, alcohol‐related unsafe driving, skipping breakfast, tanning bed use and regular consumption of fast food [15, 31, 32, 33, 34], and there is an inverse association between diet quality/healthy dietary habits and ED intake [14, 34, 35, 36].

The sociological risks of energy drink consumption in children and young people are multifaceted, and include aspects of behaviour, psychology and education. Children and young people consume energy drinks to attempt to fit in with their peers or to emulate behaviours seen across media and/or advertising [30] Lower academic performance is consistently associated with higher energy drink consumption [36], and children and young people who consume EDs are more likely to exhibit irritability and suicidal ideation [14]. There is an association between ED consumption and behaviours viewed as ‘risky′ such as use of alcohol, tobacco, and non‐prescribed drugs [37].

Previous literature highlights that consumption of EDs by children and young people was found to be patterned by gender, with boys consuming more than girls [14], and the highest consumption was observed in the most and least sedentary individuals [15, 38]. The ED industry purposely targets male audiences by associating them with extreme sports, games or any events that elicit traditional masculinities [39, 40, 41]. Other key themes have emerged which explains the reason for consumption of EDs, including the use of advertisement, taste, peer influence and physiological effects of EDs [30]. Most studies agree that consumption patterns amongst children and young people vary, however, more research is needed to understand this.

### 1.2 Current Policy and Research Context

Despite the negative health outcomes associated with EDs, sales data shows that consumption is still increasing. Many major retailers introduced a voluntary ban in 2018 following celebrity chef Jamie Oliver′s campaign called #NotForChildren [42]. In 2018, the Department of Health and Social Care ran a consultation to end the sale of EDs to under 16s in England, discussed in the green paper ‘Advancing our health: prevention in the 2020's', however there has been no further action since [43]. In July 2024, King Charles III outlined the new Labour Government's pledge to restrict the sale of high caffeine EDs to children, (under 16s) [44]. The recent 10 year Health Plan for England has promised to ban the sale of high‐caffeine energy drinks to under 16‐year‐olds [45]. The implementation of the voluntary ban in the UK has caused controversy as many retailers can continue to sell to under 16 year olds [46]. In September 2025, the UK Government announced a proposed ban on the sale of high‐caffeine energy drinks to children under 16 years old and opened a 12 week consultation. The proposal would make it illegal to sell energy drinks containing more than 150 mg of caffeine per litre to anyone under 16 years old. This applies to all retailers, including online stores, shops, restaurants, cafes, and vending machines [47].

This mixed method study aims to explore children and young people′s (in England) perceptions of EDs following the voluntary ban in 2018 and to understand their consumption patterns of EDs. The research questions were:

1. What are the current perceptions of children and young people on EDs post the 2018 voluntary ban by major retailers? (Focus groups)
2. Where, and at what cost do children and young people obtain EDs from? (Focus groups)
3. How often do children & young people consume EDs, and what influenced them to start and continue to drink them? (Questionnaire)

## 2 Methods

### 2.1 Development of the Study

We applied the Good Reporting of A Mixed Methods Study (GRAMMS) framework developed by O'Cathain, Murphy and Nicholl [48] to this study to maximise the transparency of reporting. A mixed methods research design was chosen to provide a more comprehensive understanding of this complex area than would be possible with only a quantitative or qualitative approach. Focus group questions and format were based on an earlier North East (England) qualitative study [30]. During the survey development stage, existing literature was used to draft a questionnaire; previous studies have measured ED consumption using a range of scales [35, 49, 50, 51] which makes comparison between studies challenging.

### 2.2 Public Involvement

This mixed method study involved young people in the development of the research format, tools and research questions. With the assistance of a Community Engagement Manager, an independent group of four members of the public aged 14–18 years from the North East region (England) met online for 1 hour to refine the research questions and enhance the design of a questionnaire including appropriate and user‐friendly scales to understand ED intake. Comments and suggestions were taken on board and adaptions made. Participants received a £25 voucher (payment provided by the university) for their participation in line with NIHR guidance [52].

### 2.3 Study Design

This study was conducted in June and July 2023, involved semi‐structured focus groups and an online questionnaire. We held eight semi‐structured focus groups with Years 5, 6 & 9 (9–10, 10–11, 13–14 years old) across two primary and two secondary schools in the Tees Valley Region of North East England examining young people's views on EDs after the 2018 voluntary ban. The self‐report online questionnaire was carried out with Year 9 (13–14 years old) secondary students, asking about consumption patterns of EDs alongside exploring what influences them to start and continue to consume these products.

### 2.4 Participants

These year groups were selected as previous research highlighted relatively high ED consumption [38, 53], and we aimed to explore whether the voluntary ban had impacted on availability and perceptions of energy drinks within these age groups. For the focus groups, in line with earlier work [30], Year 5 and 6 students (age 9–11 years) were identified. These children are moving towards their secondary school transition and becoming more autonomous. Year 9 students (age 13–14 years) were selected as children who may face less pressure before the start of exams and at a time when fewer young people have started experimenting with alcohol [30].

For questionnaires, students in Year 9 (age 13–14 years) were selected for the reasons above, are more autonomous and able to fill in a self‐report questionnaire [54].

Sixteen schools based in North East England were invited to participate in the study (Appendix 1); two primary and two secondary schools agreed to take part. For the focus groups, consent and assent was received from 50 parents and students. Teachers were asked to select six girls and six boys within their class at random (e.g. draw names from a hat), or if this was not appropriate, to select them diversely, for example, selecting a varied range of educational backgrounds to avoid bias, single gendered focus groups were used [30]. For the online questionnaire, consent and assent to take part was received from 22 parents and students.

### 2.5 Data Collection

Focus groups were conducted at school with one or two researchers and a teacher in the room and used to elicit in‐depth views from participants. A range of different EDs, sports and hydration drinks, and soft drink bottles were brought in for an icebreaking sorting exercise. Throughout, the format was flexible to ensure the young people felt able to discuss their thoughts. The discussion was prompted by a topic guide (Appendix 2) and audio recorded on an encrypted device (deleted within 24 hours) and transcribed verbatim. The questionnaire was an online survey (hosted by JISC Online Surveys^1^) examining ED consumption patterns; students were asked to complete this during the school day (Appendix 3).

### 2.6 Data Analysis

The data derived from the semi‐structured focus groups was analysed following principles of thematic analysis [55]. NVivo^2^ qualitative analysis software was used to systematically organise and index materials. Primary codes were identified then grouped into themes which summarised sections of data.

Questionnaire responses were transferred from Jisc Online Surveys and analysed using descriptive statistics on Excel.

### 2.7 Ethics

Informed consent was obtained from all parents or carers who were asked to opt‐in to the study if they wished their child to participate, students were required to give assent before the study began. Parents and guardians, as well as students, were given a separate participant information sheet. For the focus groups they were informed their data collected up to that point cannot be withdrawn, and for the questionnaire they could withdraw at any point during completion up to the point of data submission. Those who did not consent were not involved in the study. Ethics was granted by the University′s School of Health and Life Sciences Ethics Sub‐committee (Ref: 2023 Mar 14660).

## 3 Results

The findings from the focus groups (Years 5 and 6 (*n* = 38) and Year 9 (*n* = 12)) are presented separately from the questionnaire (Year 9 students; *n* = 22).

### 3.1 Focus Groups

Fifty children from three different schools (primary *n* = 2, secondary *n* = 1) participated in the focus groups; *n* = 38 Years 5 and 6 (9–11 years) and *n* = 12 Year 9 (13–14 years). There were three over‐arching themes (Perceptions of EDs, Motivating factors to consumption and Policy) and within these 10 sub‐themes (Branding and marketing, Ingredients, Health effects, Hydration drinks, Taste, Cost and access, Influence of friends and family, School based interventions, Age‐restricted purchase, Design and advertisement).

### 3.2 Perceptions of EDs

#### 3.2.1 Branding and Marketing

Every focus group was able to complete the sorting exercise correctly ordering drinks into soft drinks (e.g., Coca‐Cola, Sprite), energy drinks (e.g., Monster, Red Bull), and sports and hydration drinks (e.g., Lucozade sport, PRIME Hydration). Students had an awareness of different brands and a strong understanding that the brand influences behaviour:

The brand always affects the business. If I made the exact same drink, in a bottle, no one would buy it.

[Boy, Year 5/6]

Marketing of EDs came up repeatedly, including awareness of slogans (e.g. “Red Bull gives you wings”). Participants described social media (e.g. YouTube, TikTok) as platforms to market EDs, especially PRIME Hydration and Energy. Sports advertising such as F1 was described by participants, including naming Lewis Hamilton's Monster sponsorship. It was also apparent that children perceived that bright colours and designs were used to increase sales to them:

There are adverts, like you keep on watching a YouTube video and then it will pop up saying buy this.

[Girl, Year 9]

And all over YouTube and like TikTok.

[Boy, Year 5/6]

I know on F1 racing a lot of the time like Red Bull gets slapped on the side of cars, it′s a big sponsor.

Girl, Year 9]

Yeah, maybe like some of the colours because the bright might attract people I think the colours actually, I don′t know what it is, but when you taste that one, I think you′d sort of like you think of them colours.

[Girl, Year 5/6]

Participants understood that the advertisement of a computer game could influence purchase:

If I was still playing on Xbox, I probably would have took the can and got the free Xbox pass.

[Boy, Year 5/6]

#### 3.2.2 Ingredients

Children were aware of some of the ingredients within EDs, the two main ingredients being caffeine and sugar. Caffeine was highlighted as being the main difference between EDs and other beverages. However, children were uncertain about how much caffeine and sugar was in the drinks:

Energy drinks have quite a lot, I think quite a lot of caffeine in.

[Boy, Year 5/6]

Children found the ingredients list on the empty cans difficult to read, and most had not heard of the other ingredients or additives in the drinks:

T‐t‐tau‐rine, I don′t know what that is.

[Girl, Year 5/6]

You don′t really know what′s in it because things are still yet to be proven like in the soft drinks, aspartame is an ingredient and in a current study it proves you have a higher risk of developing cancer. So, you never know what some of these things actually do.

[Girl, Year 9]

#### 3.2.3 Health Effects

Children were aware of some of the negative health effects associated with drinking EDs, including addiction, insomnia, diabetes, and heart disease:

You might get like a bit addicted to it and then you might want more and more and more. And then that leads to the caffeine addiction.

[Girl, Year 5/6]

Several children also mentioned more extreme cases, from news reports or from friends or family where consumption had led to a fatality:

Someone′s died of drinking them and his mum was buying him all the bottles to collect, and she checked, because he started feeling sick and the bottles were half empty.

[Boy, Year 5/6]

#### 3.2.4 Hydration Drinks

Awareness of hydration drinks as a product (e.g., PRIME Hydration) separate to caffeinated EDs was high. Participants were keen to talk about these drinks more so than EDs. They were aware there was a difference between hydration drinks and energy drinks:

There′s two types, the one that′s hydration and the one that′s got caffeine in it.

[Boy year 5/6]

Many children perceived a positive association with PRIME Hydration and improved health effects. They described seeing children drinking hydration drinks at children's sports events like swimming, dance competitions, netball and football. However, they could not describe the positive or negative effects of the ingredients on the bottles:

I think that the PRIME is healthier for you because it′s hydration, the energy I think is a little bit more bad for you depending which one you drink, cause with the PRIME they′re all the same like they all have the same amount of things.

[Boy, Year 5/6]

They understood a lot about the promotors of the drinks (both YouTubers with links to boxing and wrestling) as well as how it is promoted, including the price variations and the hype around the drinks:

People used to punch each other over it, because it was two young famous YouTubers, Logan Paul and KSI, and they made the drinks so that people want it because it′s a drink they′ve seen all over the internet.

[Boy, Year 5/6]

So, people were paying like £10, then people have paid like £150 for one.

[Boy, Year 9]

### 3.3 Motivating Factors to Consumption

#### 3.3.1 Taste

Most highlighted taste as a motivating factor to consume EDs, they explained they were sweet and highlighted the appealing flavours:

I think the sweetness like when you drink it, you really like it because it′s really sweet.

[Girl, Year 5/6]

For PRIME Energy as well, it′s got lots of flavours like Blue Raspberry, Tropical Punch, Lemon and Lime, Watermelon, Strawberry. LOADS of flavours.

[Boy, Year 5/6]

### 3.4 Cost and Access

Participants clarified which EDs were cheaper to buy, especially the ‘own brand′ drinks. There were differing opinions on pricing and consumer choice between children. Some children explained they did not look at the cost in stores, purchasing out of brand loyalty over price. In comparison other children preferred the cheaper EDs. In addition to this, many mentioned that often ‘own brand′ EDs can be cheaper than bottled water:

No, they′re only 65p, £1. It depends on the can, like BOOST energy, cheap small can, but then you′ve got Monster £2.00 cause it′s a big brand that′s why hmm it costs more money.

[Boy, Year 5/6]

It′s at 65p, so quite cheap. Water is a bit more expensive in the shop. But I just look at the best brand.

[Girl, Year 5/6]

I would always buy the cheaper one doesn′t matter about the brand.

[Boy, Year 9]

Although many shops were listed as places to purchase EDs, participants agreed that it is easier to access EDs from a convenience store from a young age:

Yeah, yeah, a corner shop I know is getting worse now there selling them to minors. Monster and Rockstar and Red Bull to I think kids our age.

[Boy, Year 5/6]

However, shops were not the only place children had purchased EDs; they were also buying them online. A black‐market in selling drinks on was also described. They explained it is easy to buy online, where ID is not always required:

Yeah, sometimes I buy online, they don′t really check ID either.

[Girl, Year 9]

### 3.5 Influence of Friends and Family

Children suggested their friends and family were drinking EDs and that influenced them. Many used the association that they were ‘cool′ and others said that they had seen other children and young people try them to try and show off to other people:

I started seeing my friends have them and I wanted them.

[Boy, Year 5/6]

If you go to a shop and get one, sometimes it can be seen as cool.

[Girl, Year 9]

### 3.6 Policy

#### 3.6.1 School Based Interventions

While across all three schools EDs and Hydration drinks were already banned, participants suggested it would be helpful to have lessons within school about the negative health effects of ED consumption. They explained the importance of making it interactive and fun:

I think even in lesson like you can introduce a fun activity which has something to do with redesigning an energy drink, but like while doing it you can introduce different facts to raise awareness because they′re more likely to like concentrate on fun activities like that.

[Girl, Year 9]

### 3.7 Age Restricted Purchase

Across all three schools, participants assumed there was a ‘law′ on age restricted purchase but were unsure of the age (some said 10 and 13 years). They stated that shops should be stricter in selling to children, and suggested EDs should be displayed somewhere restricted:

Yeah, and if you′re going to have age ratings on things, don′t put it near the essentials like near milk and bread so they can see it, like put it away where the alcohol is, don′t make it near an area where children go.

[Boy, Year 5/6]

### 3.8 Design and Advertisement

There was consensus around the design and advertisement of EDs which was clearly perceived as being targeted and attractive to their age‐groups. Participants agreed they should change the design, colours (to make them less attractive) and increase health warning adverts:

I would make like the ones that you need ID for more dull. So it doesn′t make the kids just look at it and like ohh, that looks so cool and like buy it and try it.

[Boy, Year 5/6]

With adverts for the NHS, to say there too much sugar and that in them so if something happened to another child, they can put posters to stop people drinking them.

[Girl, Year 9]

### 3.9 Questionnaire Results

Results are presented in Tables 1 and 2. Twenty‐two Year 9 (13–14 years old) students from two secondary schools in the Tees Valley area completed the questionnaire, however, responses from those who stated they *did not* consume EDs (*n* = 4) were excluded as they did not complete the questionnaire, leaving 18 participant questionnaire responses to be analysed.

**Table 1:** Participant characteristics (*n* = 22).

Characteristic	Female	Male	(*n*)	Total %
**Energy drink consumer**				
Yes	8	10	18	81.8%
No	3	1	4	18.2%
**Age**				
13 years	2	0	2	9.0%
14 years	9	11	20	91.0%

**Table 2:** Participant questionnaire answers (*n* = 18).

Question	Female	Male	(*n*)	Total %
**What age were you when you started drinking energy drinks?**				
Under 10 years	4	1	5	27.7%
11 years	1	1	2	11.1%
12 years	2	6	8	44.4%
13 years	1	2	3	16.6%
**How often do you consume energy drinks?**				
Less than once a week	2	1	3	16.6%
Once a day, every day	0	1	1	5.5%
2–4 days a week	3	4	7	38.8%
Once a week	1	1	2	11.1%
5–6 days a week	2	3	5	22.7%
**How were you first introduced to energy drinks?**				
Advertisement	1	1	2	11.1%
At a shop	5	2	7	38.8%
By friends/family	2	3	5	22.7%
Gaming	0	1	1	5.5%
School	0	3	3	16.6%
**Where do you buy your energy drinks from?**				
Corner shop	5	4	9	50.0%
Someone else buys me it	1	1	2	11.1%
Supermarket	1	2	3	16.6%
Petrol station	2	2	4	22.2%
**Why do you choose to drink energy drinks?**				
They taste nice	5	7	12	66.6%
They give energy	1	2	3	16.6%
I like the flavours	2	1	3	16.6%
**Do you drink energy drinks when gaming?**				
Yes	0	0	0	0.0%
No	5	5	10	55.5%
Sometimes	3	5	8	44.4%
**Do you drink energy drinks when playing sport?**				
Yes	0	0	0	0.0%
No	7	4	11	61.1%
Sometimes	2	3	5	27.7%

The majority (81.8%) of children stated they consumed EDs. More males consumed EDs in comparison to females, and the data showed that most children and young people consume EDs between 2 and 4 days a week. More females appeared to be introduced to EDs at a shop, whereas males were introduced by friends and family, or at school. Respondents stated they drank EDs because they taste nice, and half bought EDs from the corner shop (convenience store) in comparison to elsewhere.

In the open‐ended questionnaire responses, some children explained that Monster, Emerge, and BOOST were their favourites to drink, while others reported they did not have a preference. Preference was influenced by the vibrant colours and flavours of EDs:

Mango loco Monster its flavourful and vibrant, I also like the pink one because I like the flavour of it.

[Girl, Year 9]

### 3.10 Hydration Drinks

Ten students (56% of the 18 that were eligible) completed the hydration and sports drinks section of the questionnaire. Those who responded ‘no′ were also excluded from the rest of the questionnaire questions.

In their open‐ended questionnaire responses children and young people reported choosing to drink hydration and sports drinks to keep hydrated, they also stated they have nice flavours and taste ‘better′ than other beverages:

They give u [sic] a boost and taste even better than just juice or something.

[Male, Year 9]

## 4 Discussion

This mixed method study, which incorporated the involvement of young people in the design, shows a complex picture of young people's perceptions of EDs and the popularity across all age groups of a relatively new product category to the market (hydration drinks) specifically, PRIME Hydration. All young people involved in the study had a strong understanding that branding and marketing is a powerful tool used to encourage purchasing of both EDs and hydration drinks. Participants enjoyed the taste and flavours of these drinks, most agreed EDs can be cheap to access, but others argued that it depends on the brand, as more widely known brands were more expensive. The consensus was that EDs and hydration drinks were easy to access at convenience stores and ‘own brand′ EDs were cheaper than water. Potential interventions to reduce purchase and consumption of EDs by young people emerged from the focus groups, restricting advertisements and product placement and making the bottles and cans less colourful. They recommended making the age restriction more obvious and to make EDs harder to purchase, such as changing the in‐store location, similar to alcohol.

This study complements previous research and highlights the importance of advertisement and branding to this age‐group. Advertisement enables ED companies to establish strong connections with their target audience, create a compelling lifestyle image, resulting in increased consumption and brand loyalty among consumers [40, 56, 57]. The importance of digital marketing to children of unhealthy products in relation to eating and health outcomes has been highlighted in a far‐reaching systematic review and metanalysis [58].

Brand loyalty and an association that brands were deemed ‘cool′ emerged from the literature multiple times and similarities can be drawn to the UK context Visram et al. study where children had the desire to look ‘cool′ to fit in with their peers [30]. Participants understood that the use of social media is a powerful marketing technique to help increase sales of EDs, whether this was the use of influencers or promotion on adverts across YouTube, Instagram or TikTok. Australian research found that energy drink brands tailor their digital content to relate to youth aspirations and social behaviours [59]. Similarities between the marketing tactics of EDs, as identified by our cohort, and alcohol marketing techniques, that is, use of social media, sponsorship of sporting and music events [60] are evident, and recent research has shown that young people are exposed to non‐permitted marketing of food and drinks via influencers on digital media [61]. Reviewing the top forty‐eight Instagram accounts of celebrities and influencers posting food and beverage advertisements, researchers found that the most frequently advertised were carbonated beverages and flavoured waters (28·5%), energy drinks (20·6%) and ready‐made foods (15·4%). This study highlights the use of highly engaging and persuasive techniques by celebrities and influencers on Instagram to target adolescents [62].

The participants described the strong brand advertising around EDs and sport, particularly Formula 1 and links to specific athletes, aligning the finding that over 90 current partnerships are with unhealthy products [63].

A key difference between the findings of the present study and previous ED research [27, 30, 32] relates to the positive attitude of the participants towards other drinks such as PRIME Hydration; a brand which also has a caffeinated product. There was awareness of the difference between the hydration and ED products. There has been no academic research around PRIME Hydration since its UK release in October 2022, but most participants spoke of this brand and product in an extremely positive way. Promoted by online influencers, there was a world‐wide social media hype about this caffeine‐free product and soon after a caffeinated version of the brand was released. While PRIME Hydration contain no added sugars, it contains artificial sweeteners such as acesulfame potassium and sucralose. Recent studies and World Health Organisation guidance have shown the negative consequences of high sweetener intake in relation to diet‐related noncommunicable diseases which are leading causes to death worldwide [24, 64, 65, 66, 67]. In addition to this, PRIME Hydration provides several vitamins which can pose a risk of vitamin toxicity if consumed in excess [68]. Although more research is still needed in this subject, the current study reflects the huge influence PRIME has had on young people within social circles.

Findings from both the focus group and questionnaire align with previous research; taste was still a key motivating factor to consumption including the sweet, appealing flavours [30, 69]. All ages and genders demonstrated awareness of different brands of EDs and their favourite flavours. Influence of family and friends toward ED consumption was also clear, which has been found in previous research as children may feel a sense of peer pressure or influence of their role models in consumption of them [1, 20]. There also appeared to be a difference between gender and consumption patterns with boys consuming more than girls, which is similar to other research as literature shows this can be down to male‐centric advertisement of products [15, 38]. Previous studies highlight cost and price discounting as a motivating factor to purchase of EDs [70, 71]; within the current research there were contrasting views as some students stated it was more about the taste and the brand which influenced purchase, whereas others reported it was simply the cost and the cheapest product.

In recent literature, gamification has been associated with increased purchase and consumption of EDs [30, 72, 73] and a link made with the aggressive promotion of energy drink within esports [74]. However, social media, adverts and influencer platforms including sponsorships were highlighted in discussions around *why* children purchase EDs. In addition to this, respondents in this study were aware of many negative health and social effects associated with the ingredients within EDs, yet previous studies report that children associated the word ‘energy′ as positive [14, 17, 75, 76]. Concurrently, previous research by Visram et al. [30] found that children and young people accessed EDs almost anywhere, however within this studies results, children and young people favoured convenience stores, as they are easier to obtain with fewer restrictions on sales compared with larger supermarkets who are more likely to enforce the voluntary restriction of sales. During the focus group discussions, children and young people described how they could purchase EDs online, a key difference from earlier research, and explained that this was an easier way to get EDs without getting ID checked. This suggests the voluntary restrictions may be having an effect or perceived effect with respect to in‐person purchases. Voluntary restrictions are stated by online retailers, however enforcement (if any) may have focused on the physical food environment and not the online food environment. This is an area which requires significant thought if there is to be legislation.

In the UK, the four devolved governments have not passed legislation on the sales of EDs to young people [43]. However, in England, a pledge to restrict the sale of high caffeine EDs to children was a key part of the Labour party's manifesto during their successful election campaign (June 2024) and a new consultation on the proposed ban opened in September 2025 [47]. Since the voluntary ban was put into place in 2018 (in major supermarkets retailers rather than convenience stores), there has been no evaluation of how effective it has been or how well, or if at all, this has been enforced. Most participants in this study thought that there was a law restricting ED sales to young people, yet they still explained they were easy to access at any age, particularly from convenience stores and online. In Visram et al. [30], children suggested that there should be an age limit for purchasing EDs but believed that this intervention could be simply overcome by asking older friends. The current study now suggests that this is accurate as young people are still able to access them underage and can also use other means (online purchasing) to obtain them. Online purchasing of EDs will be a challenge for retailers to verify age, similar to alcohol, pornography, cigarettes, fireworks, knives, non‐prescribed drugs. Participants did not highlight design and advertisement of EDs as an issue in the study by Visram et al., [30], however, within the current study, they perceived the design of the drinks as attractive to their age‐groups.

### 4.1 Limitations

This study provides new findings surrounding the perceptions of EDs and consumption patterns amongst school aged children in the UK post 2018 voluntary ban. The findings are from the North East of England, therefore may not be representative of the country as a whole. While the study tried to limit bias, there is still the possibility of some bias as despite participation being sought from schools across the Tees Valley area only three participated in the focus groups and two in the questionnaire. Teachers were asked to randomly select students to take part in the study which can potentially cause bias. The opt‐in consent could have caused bias, as it could reflect those with interests in, or concerns about EDs. In addition to this, despite trying to aim for a higher sample size for the questionnaire this proved difficult due to limited time, however there is now a co‐produced questionnaire to be used in future research.

Recruitment of schools was challenging, despite starting recruitment from April 2023; seven primary and nine secondary schools were contacted by email with little response. The recruitment areas were expanded, and 11 schools were contacted using additional personal links. Three schools dropped out with short notice, mainly due to the opt‐in processes and the need to contact parents. This can be understandable as schools are pressured especially leading up to the end of term. Once the focus groups began to run, we also ran into some complications as some children and young people were required to miss lessons, and although trying to prevent as much disruption as possible, in some groups, they were shortened to fit into the timetable. During the focus groups, every effort was made to adopt a non‐judgemental approach. Ages of the included children and young people was a limitation of the work, although this was imposed by the schools. The project ran during UK examination periods (Year 6 SATs and Year 10/11 GCSEs), and the schools understandably did not want to add this to the workload of these students. We recommend that this is taken into account in future work, and aim to run school based projects, where possible, no later than Easter.

### 4.2 Recommendations for Future Research

Recommendations for future research include working with young people to conduct field audits of their area to record the location and access of EDs to younger people. A wider study to explore if voluntary restrictions are effective or being enforced would be useful particularly at a range of outlets, supermarkets, convenience stores and online. As PRIME Hydration has become popular, it would be appropriate to gain a wider sample size to analyse consumption patterns and assess the impact on wider diet and nutrient intake, diet variety and quality.

## 5 Conclusion

This exploration of young people's perceptions of EDs and their consumption patterns has indicated a multifaceted situation. The interplay of marketing strategies, brand association, individual preferences, such as taste, price, and brand, as well as peer influence contributes to the complexity around how these beverages are perceived and consumed by young people. More participants were consuming EDs than they were choosing to abstain highlighting easy access to EDs despite the voluntary ban being in place. The findings highlight that there is not a sole determinant for increased ED consumption in this age‐group, yet the power of advertisement and promotion should not be underestimated. More importantly, participants had a huge positive association with PRIME Hydration, yet little research has been done on the health effects of this product. It is crucial to acknowledge that further research is needed to fully grasp the implications and potential consequences of ED and hydration drink use and involving young people in further research is fundamental for ultimately guiding responsible policy making that prioritises their health.

## Author Contributions

**Grace Stewart:** main author, assisted in formulating the research questions based on researching previous literature, designing the study type, carried out the studies and sorting resources for qualitative group work, analysed the data using descriptive analysis and NVivo, interpreted the findings, developed the concept and design of the article, and wrote the article. **Amelia A Lake:** corresponding author, formulated the research questions, study design, conducted data collection, supervision of G.S., interpreted the findings, developed the concept and design of the article, and wrote the article. **Helen J Moore:** ethics lead, formulated the research questions, study design, supervision of G.S., interpreted the findings, developed the concept and design of the article, and wrote the article. All authors have reviewed and approved the final submitted version of the manuscript.

## Ethics Statement

Ethics was granted by the University′s School of Health and Life Sciences Ethics Sub‐committee (Ref: 2023 Mar 14660).

## Conflicts of Interest

The authors declare no conflicts of interest.

## Peer Review

The peer review history for this article is available at https://www.webofscience.com/api/gateway/wos/peer-review/10.1111/jhn.70140.

## Transparency Declaration

Lake is Deputy Director of Fuse the Centre for Translational Research in Public Health and is Professor of Public Health Nutrition at Teesside University. She sits on the scientific committee of the British Nutrition Foundation and is an executive for Nutrition North (Northern Health Science Alliance). All authors have no industry associations, or involvement with any of the mentioned brands.

## Supporting information

Appendix 1‐ School recruitment.

Appendix 2 ‐ Topic guide.

Appendix 3 ‐ JISC Online Surveys Questionnaire.

## Data Availability

The data that support the findings of this study are available from the corresponding author upon reasonable request.

## Endnotes

1
www.jisc.ac.uk/online-surveys

2
www.lumivero.com/products/nvivo/

## References

[1] S. Visram , M. Cheetham , D. M. Riby , S. J. Crossley , and A. A. Lake , “Consumption of Energy Drinks by Children and Young People: A Rapid Review Examining Evidence of Physical Effects and Consumer Attitudes,” BMJ Open 6, no. 10 (2016): e010380. 10.1136/bmjopen-2015-010380 PMC5073652 27855083

[2] H. Ariffin , X. Q. Chong , P. N. Chong , and P. N. Okechukwu , “Is the Consumption of Energy Drink Beneficial or Detrimental to Health: A Comprehensive Review?,” Bulletin of the National Research Centre 46, no. 1 (2022): 163.

[3] B. Campbell , C. Wilborn , P. La Bounty , et al., “International Society of Sports Nutrition Position Stand: Energy Drinks,” Journal of the International Society of Sports Nutrition 10, no. 1 (2013): 2171314. 10.1186/1550-2783-10-1 PMC3538552 23281794

[4] A. Grumezescu and A. M. Holban , “Sports and Energy Drinks.” Science of Beverages (Elsevier Science, 2019). 10.

[5] C. M. Reyes and M. C. Cornelis , “Caffeine in the Diet: Country‐Level Consumption and Guidelines,” Nutrients 10, no. 11 (2018): 1772. 30445721 10.3390/nu10111772 PMC6266969

[6] K. H. Lee , G. Human , J. Fourie , W. Louw , C. Larson , and G. Joubert , “Medical Students' Use of Caffeine for ‘Academic Purposes′ and Their Knowledge of Its Benefits, Side‐Effects and Withdrawal Symptoms,” South African Family Practice 51, no. 4 (2009): 322–327.

[7] J. L. Temple , C. Bernard , S. E. Lipshultz , J. D. Czachor , J. A. Westphal , and M. A. Mestre , “The Safety of Ingested Caffeine: A Comprehensive Review,” Frontiers in Psychiatry 8 (2017): 80. 28603504 10.3389/fpsyt.2017.00080 PMC5445139

[8] J. O. Brower and J. L. Swatek , “Beyond the Buzz: The Fatal Consequences of Caffeine Overconsumption,” Journal of Analytical Toxicology 48, no. 8 (2024): 535–540. 38814665 10.1093/jat/bkae046

[9] C. L. Gardiner , J. Weakley , L. M. Burke , et al., “Dose and Timing Effects of Caffeine on Subsequent Sleep: A Randomized Clinical Crossover Trial,” Sleep 48, no. 4 (2025): zsae230. 39377163 10.1093/sleep/zsae230 PMC11985402

[10] European Food Safety Authority . Scientific opinion on the safety of caffeine 2015. https://www.efsa.europa.eu/sites/default/files/consultation/150115.pdf.

[11] V. S. Malik , A. Pan , W. C. Willett , and F. B. Hu , “Sugar‐Sweetened Beverages and Weight Gain in Children and Adults: A Systematic Review and Meta‐Analysis,” American Journal of Clinical Nutrition 98, no. 4 (2013): 1084–1102. 23966427 10.3945/ajcn.113.058362 PMC3778861

[12] A. V. Mattioli , A. Manenti , and A. Farinetti , “Energy Drinks and Heart Damage in Young People,” Journal of Tehran Heart Center 17, no. 4 (2022): 255–256. 37143749 10.18502/jthc.v17i4.11617 PMC10154118

[13] S. M. Seifert , J. L. Schaechter , E. R. Hershorin , and S. E. Lipshultz , “Health Effects of Energy Drinks on Children, Adolescents, and Young Adults,” Pediatrics 127, no. 3 (2011): 511–528. 21321035 10.1542/peds.2009-3592 PMC3065144

[14] C. Ajibo , A. Van Griethuysen , S. Visram , and A. A. Lake , “Consumption of Energy Drinks by Children and Young People: A Systematic Review Examining Evidence of Physical Effects and Consumer Attitudes,” Public Health 227 (2024): 274–281. 38228408 10.1016/j.puhe.2023.08.024

[15] M. Puupponen , J. Tynjälä , R. Välimaa , and L. Paakkari , “Associations Between Adolescents' Energy Drink Consumption Frequency and Several Negative Health Indicators,” BMC Public Health 23, no. 1 (2023): 258. 36747163 10.1186/s12889-023-15055-6 PMC9903583

[16] M. A. Heckman , J. Weil , and E. G. de Mejia , “Caffeine (1, 3, 7‐trimethylxanthine) in Foods: A Comprehensive Review on Consumption, Functionality, Safety, and Regulatory Matters,” Journal of Food Science 75, no. 3 (2010): 77–87. 10.1111/j.1750-3841.2010.01561.x 20492310

[17] M. Marinoni , M. Parpinel , A. Gasparini , M. Ferraroni , and V. Edefonti , “Risky Behaviors, Substance Use, and Other Lifestyle Correlates of Energy Drink Consumption in Children and Adolescents: A Systematic Review,” European Journal of Pediatrics 181, no. 4 (2022): 1307–1319. 34988663 10.1007/s00431-021-04322-6

[18] M. Rostami , M. Babashahi , S. Ramezani , and H. Dastgerdizad , “A Scoping Review of Policies Related to Reducing Energy Drink Consumption in Children,” BMC Public Health 24, no. 1 (2024): 2308. 39187818 10.1186/s12889-024-19724-y PMC11346296

[19] D. Nowak and A. Jasionowski , “Analysis of the Consumption of Caffeinated Energy Drinks Among Polish Adolescents,” International Journal of Environmental Research and Public Health 12, no. 7 (2015): 7910–7921. 26184263 10.3390/ijerph120707910 PMC4515699

[20] A. A. Alsunni , “Energy Drink Consumption: Beneficial and Adverse Health Effects,” International Journal of Health Sciences 9, no. 4 (2015): 459–465. PMC4682602 26715927

[21] A. V. Mattioli , “Energy Drinks and Obesity: Preliminary Results From a Preclinical Study,” Anatolian Journal of Cardiology 19, no. 6 (2018): 422. 29848927 10.14744/AnatolJCardiol.2018.25826 PMC5998865

[22] A. J. Jeffers , K. E. Vatalaro Hill , and E. G. Benotsch , “Energy Drinks, Weight Loss, and Disordered Eating Behaviors,” Journal of American College Health 62, no. 5 (2014): 336–342. 24635529 10.1080/07448481.2014.902838

[23] J. M. Marino , T. E. Ertelt , S. A. Wonderlich , et al., “Caffeine, Artificial Sweetener, and Fluid Intake in Anorexia Nervosa,” International Journal of Eating Disorders 42, no. 6 (2009): 540–545. 19189405 10.1002/eat.20633 PMC3022267

[24] H. Li , H. Liang , H. Yang , et al., “Association Between Intake of Sweetened Beverages With All‐Cause and Cause‐Specific Mortality: A Systematic Review and Meta‐Analysis,” Journal of Public Health 44, no. 3 (2022): 516–526. 33837431 10.1093/pubmed/fdab069

[25] S. Mirfarsi , Y. Lin , S. Dadjoo , H.‐H. Sun , and J. A. Elo , “The Impact of Sugar‐Sweetened Beverages on Type 2 Diabetes and Obesity and the Role of Oral Health Care Providers,” Journal of the California Dental Association 48, no. 11 (2020): 603–609.

[26] D. Wiggers , J. L. Reid , C. M. White , and D. Hammond , “Use and Perceptions of Caffeinated Energy Drinks and Energy Shots in Canada,” American Journal of Preventive Medicine 53, no. 6 (2017): 866–871. 28755982 10.1016/j.amepre.2017.05.021

[27] C. Vogel , S. Shaw , S. Strömmer , et al., “Inequalities in Energy Drink Consumption Among UK Adolescents: A Mixed‐Methods Study,” Public Health Nutrition 26, no. 3 (2023): 575–585. 36472075 10.1017/S1368980022002592 PMC9989712

[28] C. Khouja , D. Kneale , G. Brunton , et al., “Consumption and Effects of Caffeinated Energy Drinks in Young People: An Overview of Systematic Reviews and Secondary Analysis of UK Data to Inform Policy,” BMJ Open 12, no. 2 (2022): e047746. 10.1136/bmjopen-2020-047746 PMC8830236 35131813

[29] Statista . Energy drinks market in the UK—statistics & facts 2024. https://www.statista.com/topics/11676/energy-drinks-in-the-united-kingdom/.

[30] S. Visram , S. J. Crossley , M. Cheetham , and A. Lake , “Children and Young People's Perceptions of Energy Drinks: A Qualitative Study,” PLoS One 12, no. 11 (2017): e0188668. 29190753 10.1371/journal.pone.0188668 PMC5708842

[31] N. Larson , J. DeWolfe , M. Story , and D. Neumark‐Sztainer , “Adolescent Consumption of Sports and Energy Drinks: Linkages to Higher Physical Activity, Unhealthy Beverage Patterns, Cigarette Smoking, and Screen Media Use,” Journal of Nutrition Education and Behavior 46, no. 3 (2014): 181–187. 24809865 10.1016/j.jneb.2014.02.008 PMC4023868

[32] A. O. Markon , M. Ding , J. E. Chavarro , and B. J. Wolpert , “Demographic and Behavioural Correlates of Energy Drink Consumption,” Public Health Nutrition 26 (2022): 1424–1435. 36214079 10.1017/S1368980022001902 PMC10258655

[33] C. Protano “Consumption Patterns of Energy Drinks in University Students: A Systematic Review and Meta‐analysis.” 2022. https://www.sciencedirect.com/science/article/pii/S0899900722003161. 10.1016/j.nut.2022.111904 36529090

[34] Z. D. Veselska , D. Husarova , and M. Kosticova , “Energy Drinks Consumption Associated With Emotional and Behavioural Problems via Lack of Sleep and Skipped Breakfast Among Adolescents,” International Journal of Environmental Research and Public Health 18, no. 11 (2021): 6055. 34199877 10.3390/ijerph18116055 PMC8200076

[35] S. Y. Kim , S. Sim , and H. G. Choi , “High Stress, Lack of Sleep, Low School Performance, and Suicide Attempts Are Associated With High Energy Drink Intake in Adolescents,” PLoS One 12, no. 11 (2017): e0187759. 29135989 10.1371/journal.pone.0187759 PMC5685612

[36] A. P. Smith and G. Richards , “Energy Drinks, Caffeine, Junk Food, Breakfast, Depression and Academic Attainment of Secondary School Students,” Journal of Psychopharmacology 32, no. 8 (2018): 893–899. 29947575 10.1177/0269881118783314

[37] A. Dawodu and K. Cleaver , “Behavioural Correlates of Energy Drink Consumption Among Adolescents: A Review of the Literature,” Journal of Child Health Care 21, no. 4 (2017): 446–462. 29110525 10.1177/1367493517731948

[38] S. Visram , M. Cheetham , D. Riby , A. Lake , and S. Crossley , “A Systematic Review of the Effects Associated With Children and Young People's Use of Energy Drinks,” European Journal of Public Health 25, no. 3 (2015): 70.

[39] K. E. Miller , “Wired: Energy Drinks, Jock Identity, Masculine Norms, and Risk Taking,” Journal of American College Health 56, no. 5 (2008): 481–490. 18400659 10.3200/JACH.56.5.481-490 PMC2562885

[40] C. A. Goodhew , T. L. Perry , and N. J. Rehrer , “Factors Influencing Energy Drink Consumption in Participants and Viewers of Extreme Sports,” Journal of Nutrition and Metabolism 7 (2020): 9382521. 10.1155/2020/9382521 PMC7563047 33083056

[41] D. Hammond and J. L. Reid , “Exposure and Perceptions of Marketing for Caffeinated Energy Drinks Among Young Canadians,” Public Health Nutrition 21, no. 3 (2018): 535–542. 29151382 10.1017/S1368980017002890 PMC10260760

[42] R. Smithers UK supermarkets ban sales of energy drinks to under‐16s 2018 [cited 2022 28 December]. https://www.theguardian.com/lifeandstyle/2018/mar/05/uk-supermarkets-ban-sales-energy-drinks-under-16s.

[43] HM Government . Advancing our health: prevention in the 2020s 2019. https://assets.publishing.service.gov.uk/government/uploads/system/uploads/attachment_data/file/819766/advancing-our-health-prevention-in-the-2020s-accessible.pdf.

[44] UK Government . Oral statement to Parliament: The King′s Speech 2024. 2024.

[45] HM Government . 10 Year Health Plan for England: fit for the future. London; 2025 03‐07‐2025.

[46] ACS . The voice of local shops 2023. https://www.acs.org.uk/sites/default/files/energy-drinks-guideforretailers.pdf.

[47] Department of Health and Social Care, Department for Education . Ban on selling high‐caffeine energy drinks to boost kids' health. In: Education DoHaSCDf, editor. 2025.

[48] A. O'cathain , E. Murphy , and J. Nicholl , “The Quality of Mixed Methods Studies in Health Services Research,” Journal of Health Services Research & Policy 13, no. 2 (2008): 92–98. 10.1258/jhsrp.2007.007074 18416914

[49] K. E. Miller , K. H. Dermen , and J. F. Lucke , “Caffeinated Energy Drink Use by U.S. Adolescents Aged 13–17: A National Profile,” Psychology of Addictive Behaviors 32, no. 6 (2018): 647–659. 30124307 10.1037/adb0000389 PMC6136946

[50] H. Sampasa‐Kanyinga , H. A. Hamilton , and J. P. Chaput , “Sleep Duration and Consumption of Sugar‐Sweetened Beverages and Energy Drinks Among Adolescents,” Nutrition 48 (2018): 77–81. 29469025 10.1016/j.nut.2017.11.013

[51] S. Scuri , F. Petrelli , M. Tesauro , F. Carrozzo , L. Kracmarova , and I. Grappasonni , “Energy Drink Consumption: A Survey in High School Students and Associated Psychological Effects,” Journal of Preventive Medicine and Hygiene 59, no. 1 (2018): 75. 10.15167/2421-4248/jpmh2018.59.1.898 PMC6009064 29938241

[52] National Institute for Health and Care Research . Payment guidance for researchers and professionals 2024. http://www.nihr.ac.uk/payment-guidance-researchers-and-professionals.

[53] S. Kaldenbach , T. A. Strand , B. S. Solvik , and M. Holten‐Andersen , “Social Determinants and Changes in Energy Drink Consumption Among Adolescents in Norway, 2017–2019: a Cross‐Sectional Study,” BMJ Open 11, no. 8 (2021): e049284. 10.1136/bmjopen-2021-049284 PMC8381306 34417216

[54] P. Patalay , D. Hayes , and M. Wolpert , “Assessing the Readability of the Self‐Reported Strengths and Difficulties Questionnaire,” BJPsych Open 4, no. 2 (2018): 55–57. 29971145 10.1192/bjo.2017.13 PMC6020258

[55] V. Braun and V. Clarke , “Thematic analysis.” Encyclopedia of Quality of Life and Well‐Being Research (Springer, 2024), 7187–7193.

[56] É. Demers‐Potvin , M. White , M. Potvin Kent , et al., “Adolescents' Media Usage and Self‐Reported Exposure to Advertising Across Six Countries: Implications for Less Healthy Food and Beverage Marketing,” BMJ Open 12, no. 5 (2022): e058913. 10.1136/bmjopen-2021-058913 PMC9121490 35589343

[57] E. Kay , E. Kemps , I. Prichard , and M. Tiggemann , “Instagram‐Based Priming to Nudge Drink Choices: Subtlety is Not the Answer,” Appetite 180 (2023): 106337. 36210015 10.1016/j.appet.2022.106337

[58] E. Boyland , M. Muc , A. Coates , et al., “Food Marketing, Eating and Health Outcomes in Children and Adults: A Systematic Review and Meta‐Analysis,” British Journal of Nutrition 133, no. 6 (2025): 781–805. 40518855 10.1017/S0007114524000102 PMC12169957

[59] L. Buchanan , H. Yeatman , B. Kelly , and K. Kariippanon , “A Thematic Content Analysis of How Marketers Promote Energy Drinks on Digital Platforms to Young Australians,” Australian and New Zealand Journal of Public Health 42, no. 6 (2018): 530–531. 30370962 10.1111/1753-6405.12840

[60] D. Jernigan and C. S. Ross , “The Alcohol Marketing Landscape: Alcohol Industry Size, Structure, Strategies, and Public Health Responses,” Journal of Studies on Alcohol and Drugs, Supplement 19, no. 19 (2020): 13–25. 10.15288/jsads.2020.s19.13 PMC7064002 32079559

[61] B. Naderer , M. Wakolbinger , S. Haider , et al., “Influencing Children: Food Cues in Youtube Content From Child and Youth Influencers,” BMC Public Health 24, no. 1 (2024): 3340. 39614227 10.1186/s12889-024-20870-6 PMC11607801

[62] R. Khan , A. Tanweer , and L. S. Suggs , “Analysing Celebrity and Influencer Marketing of Food and Beverages to Adolescents on Instagram,” Public Health Nutrition 28, no. 1 (2025): e144. 40859419 10.1017/S1368980025101006 PMC12516620

[63] S. Borland , “Junk Food ‘Avoids Advertising Regulation’ With Top Level UK Sports Sponsorship,” BMJ 390 (2025): r1363. 40633966 10.1136/bmj.r1363

[64] C. Debras , E. Chazelas , L. Sellem , et al., “Artificial Sweeteners and Risk of Cardiovascular Diseases: Results From the Prospective Nutrinet‐Santé Cohort,” BMJ 378 (2022): e071204. 36638072 10.1136/bmj-2022-071204 PMC9449855

[65] K. R. Tandel , “Sugar Substitutes: Health Controversy Over Perceived Benefits,” Journal of Pharmacology and Pharmacotherapeutics 2, no. 4 (2011): 236–243. 22025850 10.4103/0976-500X.85936 PMC3198517

[66] World Health Organization . Use of non‐sugar sweeteners: WHO guideline 2023. https://www.who.int/publications/i/item/9789240073616. 37256996

[67] Z. Chen , C. Wei , S. Lamballais , et al., “Artificially Sweetened Beverage Consumption and All‐Cause and Cause‐Specific Mortality: An Updated Systematic Review and Dose‐Response Meta‐Analysis of Prospective Cohort Studies,” Nutrition Journal 23, no. 1 (2024): 86. 39085903 10.1186/s12937-024-00985-7 PMC11290234

[68] S. Remmer PRIME Hydration: Is it safe for kids? 2023. https://www.sarahremmer.com/prime-hydration-is-it-safe-for-kids/#:~:text=For%20older%20kids%2C%20Prime%20Hydration,age%20of%2018%20years%20old.

[69] S. Champlin and K. Pasch , Reasons for Energy Drink Consumption Among Ethnically Diverse High School Students (Boston, MA: APHA, 2013).

[70] H. Mamiya , E. E. M. Moodie , A. M. Schmidt , Y. Ma , and D. L. Buckeridge , “Price Discounting as a Hidden Risk Factor of Energy Drink Consumption,” Canadian Journal of Public Health 112, no. 4 (2021): 638–646. 33725331 10.17269/s41997-021-00479-7 PMC8225783

[71] B. Nelson , M. Giveans , C. Anderson , and R. Johnson , “Characteristics of Energy Drink Consumption in High‐School Football Players,” Clinical Journal of Sport Medicine 18, no. 2 (2008): 1.

[72] A. Jain , “In‐Game Advertising and Brand Purchase Intentions: An SOR Perspective,” Global Knowledge, Memory and Communication 1 (2022): 1.

[73] C. Y. Yang , F. C. Chang , R. Rutherford , et al., “Excessive Gaming and Online Energy‐Drink Marketing Exposure Associated With Energy‐Drink Consumption Among Adolescents,” International Journal of Environmental Research and Public Health 19, no. 17 (2022): 10661. 36078377 10.3390/ijerph191710661 PMC9518090

[74] G. Kerr , H. Pu , J. Dalton , and C. Daprano , “Gaming for Fuel: A Content Analysis of Energy Drink Advertising in Esports,” Performance Enhancement & Health 13, no. 4 (2025): 100351.

[75] H. Bunting , A. Baggett , and J. Grigor , “Adolescent and Young Adult Perceptions of Caffeinated Energy Drinks. A Qualitative Approach,” Appetite 65 (2013): 132–138. 23419966 10.1016/j.appet.2013.02.011

[76] J. A. O'Dea , “Consumption of Nutritional Supplements Among Adolescents: Usage and Perceived Benefits,” Health Education Research 18, no. 1 (2003): 98–107. 12608687 10.1093/her/18.1.98

