# Supplementary material for: A Mixed Method Study Exploring Children and Young People's Perception of Energy Drinks and Analysing Consumption Patterns
Source: J Hum Nutr Diet. 2025 Oct 27;38(5):e70140. doi: 10.1111/jhn.70140 (PMC12557189; doi:10.1111/jhn.70140)
Supplement: Supplementary file 1 — Appendix 1‐ School recruitment. [file JHN-38-0-s003.docx]

**Appendix 1- School recruitment**

| **School** | **How they were approached** | **Outcome** |
| --- | --- | --- |
| Primary school 1 and 2 | Recruited by personal link to school | Confirmed to participate in the study |
| Primary school 3 to 7 | Two emails sent to try recruit | No responses, did not participate in the study |
| Secondary school 1 | Recruited by personal link to school | Confirmed to participate in the study |
| Secondary school 2 | Recruited by personal link to school | School changed the date twice, up until the end of term therefore only able to do survey |
| Secondary school 3 | Several emails sent to encourage recruitment | A school did not feel comfortable sending out the consent forms and PIS to all Year 9 pupils parents for a number of reasons they did not list so had to rely on volunteers (no one volunteered) |
| Secondary school 4 | Two emails sent to recruit | Consent forms were sent out and children did not return them |
| Secondary school 5-9 | Two emails sent to recruit | No response, did not participate in the study |
| 11 other external links | Email sent to encourage recruitment | Although people were trying to help to gain support, there was lack of interest from schools |
