# Supplementary material for: A Mixed Method Study Exploring Children and Young People's Perception of Energy Drinks and Analysing Consumption Patterns
Source: J Hum Nutr Diet. 2025 Oct 27;38(5):e70140. doi: 10.1111/jhn.70140 (PMC12557189; doi:10.1111/jhn.70140)
Supplement: Supplementary file 2 — Appendix 2 ‐ Topic guide. [file JHN-38-0-s001.docx]

**Appendix 2- Topic guide**

Time: Up to an hour

Resources: Give out PIS, gain assent

| **Discussion categories** | **Discussion themes** |
| --- | --- |
| Introduction | Welcome and introduction |
| Ground rules | Right to withdraw  Group etiquette  Privacy/confidentiality/anonymity  Use of voice recording |
| **Topics guide for EDs discussion** | |
| What is meant by ‘energy drinks’ | Sorting exercise with different drinks- sports drink, hydration drink/energy drink/soft drink  Ingredients |
| Do children drink energy drinks in school or around your neighbourhood | Any patterns, by age, gender, pupil characteristics  Trends over time |
| Does anyone recognise any familiar brands | Cost  Awareness of branding and marketing  Gaming |
| How do children access energy drinks | When, where and how they obtain, or consume energy drinks  Why do children choose to drink them  Risks to drinking them |
| What are the benefits/harms of energy drinks | Health and wellbeing  Behaviour  Education  Social effects |
| What interventions might stop young people from buying and drinking them | School  Age restricted purchases  Labelling and marketing |
| Closing statements | Further comments/questions  Thank everyone  Offer any contact post-focus group if individuals want to debrief |
