# Supplementary material for: A Mixed Method Study Exploring Children and Young People's Perception of Energy Drinks and Analysing Consumption Patterns
Source: J Hum Nutr Diet. 2025 Oct 27;38(5):e70140. doi: 10.1111/jhn.70140 (PMC12557189; doi:10.1111/jhn.70140)
Supplement: Supplementary file 3 — Appendix 3 ‐ JISC Online Surveys Questionnaire. [file JHN-38-0-s002.docx]

**Appendix 3 - JISC Online Surveys Questionnaire**

**Energy Drinks, Children and Young People**

Introduction

**Energy Drinks, Children and Young People**

**Energy Drinks (Energy drinks are beverages that typically contain caffeine and sugar or sweetener)**

Currently, do you drink energy drinks?

Yes

No

**Energy Drinks (Energy drinks are beverages that typically contain caffeine and sugar or sweetener)**

How often do you consume an energy drink?

Never

Less than once a week

Once a week

2-4 days a week

5-6 days a week

Once a day, every day

Every day, more than once

What age were you when you first had an energy drink?

Under 10 years

11 years

12 years

13 years

14 years

How were you first introduced to energy drinks?

By friends/family

At a shop

Gaming

School

Advertisement (please say where in box below)

Other (please say in box below)

If you selected Other, please specify:

Where do you buy your energy drinks from? (select as many as appropriate)

Supermarket

Corner shop

Petrol station

Someone else buys it

Other (please tell us more)

If you selected Other, please tell us more:

Why do you choose you drink energy drinks? (please tell us more about this)

Which energy drink do you prefer the most and why? (please tell us more about this)

Do you drink energy drinks when gaming?

Yes

No

Sometimes

Do you drink energy drinks when playing sport?

Yes

No

Sometimes

**Sport/hydration drinks (functional beverages whose stated purpose is to help replace water, electrolytes, and energy before, during and especially after training)**

Currently, do you drink hydration or sports drinks?

Yes

No

Sometimes

**Sport/hydration drinks (functional beverages whose stated purpose is to help replace water, electrolytes, and energy before, during and especially after training)**

Why do you choose to drink sports/hydration drinks? (please tell us more)

Which hydration or sports drinks do you choose to drink and why? (please tell us more)

How were you first introduced to hydration/sports drinks? (please choose one)

By friends/family

At a shop

Gaming

School

Advertisement (please say where in box below)

Other

If you selected Other, please specify:

**Details about you**

Your Gender

Male

Female

Prefer not to say

Other (please say)

If you selected Other, please specify:

Your Age

**Final page**

Thank you for taking the time to complete this survey.

Bottom of Form
